# Signatures of tumor-associated macrophages correlate with treatment response in ovarian cancer patients

**DOI:** 10.18632/aging.205362

**Published:** 2024-01-03

**Authors:** Yang Gao, Yuwen Qi, Yin Shen, Yaxing Zhang, Dandan Wang, Min Su, Xuelian Liu, Anjin Wang, Wenwen Zhang, Can He, Junyuan Yang, Mengyuan Dai, Hua Wang, Hongbing Cai

**Affiliations:** 1 Department of Gynecological Oncology, Zhongnan Hospital of Wuhan University, Wuhan, China; 2 Hubei Key Laboratory of Tumor Biological Behaviors, Wuhan, China; 3 Hubei Cancer Clinical Study Center, Wuhan, China; 4 Department of Integrative Ultrasound Medicine, Zhongnan Hospital of Wuhan University, Wuhan, China

**Keywords:** ovarian cancer (OC), tumor-associated macrophages (TAMs), immunotherapy, treatment

## Abstract

Ovarian cancer (OC) ranks as the second leading cause of death among gynecological cancers. Numerous studies have indicated a correlation between the tumor microenvironment (TME) and the clinical response to treatment in OC patients. Tumor-associated macrophages (TAMs), a crucial component of the TME, exert influence on invasion, metastasis, and recurrence in OC patients. To delve deeper into the role of TAMs in OC, this study conducted an extensive analysis of single-cell data from OC patients. The aim is to develop a new risk score (RS) to characterize the response to treatment in OC patients to inform clinical treatment. We first identified TAM-associated genes (TAMGs) in OC patients and examined the protein and mRNA expression levels of TAMGs by Western blot and PCR experiments. Additionally, a scoring system for TAMGs was constructed, successfully categorizing patients into high and low RS subgroups. Remarkably, significant disparities were observed in immune cell infiltration and immunotherapy response between the high and low RS subgroups. The findings revealed that patients in the high RS group had a poorer prognosis but displayed greater sensitivity to immunotherapy. Another important finding was that patients in the high RS subgroup had a higher IC50 for chemotherapeutic agents. Furthermore, further experimental investigations led to the discovery that THEMIS2 could serve as a potential target in OC patients and is associated with EMT (epithelial-mesenchymal transition). Overall, the TAMGs-based scoring system holds promise for screening patients who would benefit from therapy and provides valuable information for the clinical treatment of OC.

## INTRODUCTION

Ovarian cancer (OC) stands as one of the most pernicious malignancies afflicting the female reproductive system. It is distinguished by an absence of discernible early symptoms and an insufficiency of reliable methods for early detection [1, 2]. Notwithstanding notable strides in the treatment of OC, particularly in the burgeoning realm of immunotherapies, the outlook for OC patients remains disheartening, with an approximate 80% fatality rate among individuals afflicted by advanced ovarian cancer.

The tumor microenvironment assumes a paramount role in the context of OC, with macrophages serving as a significant constituent thereof. These macrophages, known as tumor-associated macrophages (TAMs), originate from peripheral blood mononuclear cells and infiltrate solid cancer tissues, making substantial contributions to their growth [3–5]. TAMs actively participate in various stages of ovarian cancer progression, including immune evasion, migration, metastasis, and angiogenesis. For instance, Mingzhu et al. conducted a study revealing that TAMs can accelerate spheroid formation and promote metastasis during the early stages of OC [6]. Additionally, TAMs secrete exosomes containing small RNA molecules, which can be internalized by ovarian cancer cells and enhance their resistance to drugs [7]. TAMs emerge as pivotal constituents of the OC microenvironment, conceivably holding sway over the clinical response to OC [8, 9]. They serve as vital factors in categorizing OC patients into “hot” and “cold” tumors. Consequently, further investigations focusing on TAMs in OC patients are warranted.

**Figure 1:**
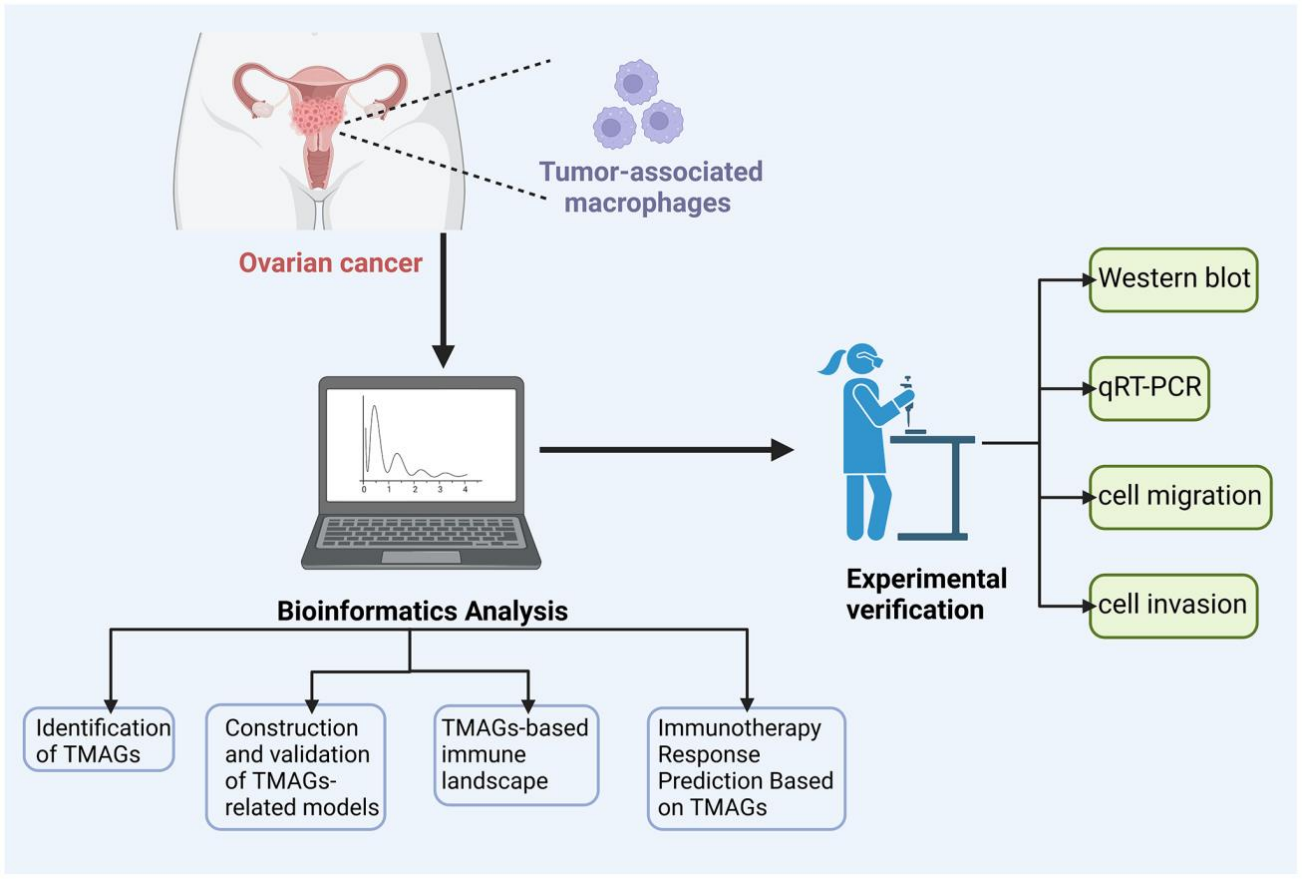
Flow chart.

In this study, our initial focus was on identifying TAM-related genes (TAMGs) specific to OC through the analysis of single-cell data. Subsequently, we conducted further screening to pinpoint the key TAMGs. Building upon this, we developed a scoring system based on these genes to evaluate TAM-related activity and differentiate between OC patients. Remarkably, patients with high and low TAM scores exhibited substantial differences in their prognostic status and response to immunotherapy and drug chemotherapy. Acknowledging the significant heterogeneity among cancer patients, our scoring system holds promise for facilitating more personalized treatment approaches. To illustrate the research workflow, please refer to Figure 1 in our study.

## METHODS

### OC’s data collection and collation

The Cancer Genome Atlas (TCGA) database, accessible at https://portal.gdc.cancer.gov/, afforded us access to a comprehensive repository comprising 379 samples procured from 376 OC patients. In order to establish an appropriate control group consisting of non-cancer patients, we harnessed data sourced from the Genotype-Tissue Expression (GTEx) project, available at https://gtexportal.org/home [10, 11]. To facilitate uniformity in our analyses, we standardized the expression values across various datasets to Transcripts per Kilobase of exon models per Million mapped reads TPM) values [12, 13]. In addition, we applied the “ComBat” algorithm to effectively mitigate potential (batch effects stemming from non-biotechnology-related biases inherent to the datasets.

For our investigation of single-cell RNA sequencing data, we curated datasets GSE115007 and GSE118828 from the Geo database, which can be accessed at https://www.ncbi.nlm.nih.gov/geo/ [14, 15]. Furthermore, we incorporated the GSE63885 dataset as an auxiliary resource and validation set for the TCGA data. The GSE63885 dataset encompasses gene expression profiles derived from 101 surgical samples of ovarian cancer [16].

To bolster our analysis, the Cancer Immunome Database (TCIA), available at https://tcia.at/home, furnished us with the Immunophenoscore (IPS). The IPS, a robust predictive tool for assessing the responsiveness to immunotherapy targeting CTLA-4 and PD-1, played a pivotal role in our study [17].

### Cell culture and transfection

The IOSE-80 human normal ovarian cell line was obtained from Yaji Biological (Shanghai, China), while the A2780 and SKOV-3 cell lines were procured from Procell (Wuhan, China). A2780 cells were cultured in RPMI-1640 medium (Procell) supplemented with 2 mM glutamine (Sangon Biotech, Shanghai, China) and 10% fetal bovine serum (FBS, Gibco). IOSE-80 and SKOV-3 cells were cultured in RPMI-1640 medium containing 10% FBS. All cells were maintained in a CO2 incubator at 37°C. siRNAs targeting human THEMIS2 (siRNA A: caauguguacagcaagauu, siRNA B: gaucccgucuacgcuggauu) were procured from Dharmacon (Epson, UK). Transfection of cells was carried out using Lipofectamine RNAiMAX (Invitrogen, USA) in accordance with the manufacturer's instructions.

### Identification of TAMGs

In Tumor Immune Single-cell Hub 2 (TISCH2), tumor immunology and microenvironment are addressed through a scRNA-seq database [18]. To identify key TAMGs, we first screened for shared genes by extracting TAMGs from two independent OC single-cell data (GSE115007 and GSE118828) via the TISCH2 database. We then performed further screening on these genes’ expression levels and prognosis in OC (TCGA-OV and GSE63885). TAMGs were genes with significant dysregulation and significant correlation with prognosis in the OC group compared to the normal group. In addition, two different algorithms (EPIC and TIMER) were used to further validate the association of genes with TAMs. The GEPIA database was used to validate the mRNA expression levels of TAMGs between the normal and OC groups [19]. TAMG protein expression levels were investigated further using the Human Protein Atlas (HPA) database [20, 21].

### Quantitative real-time PCR (qRT-PCR)

Total RNA was extracted from the cells using Trizol (Invitrogen, USA) according to the manufacturer’s protocol. The extracted RNA was then reverse-transcribed into cDNA using the Reverse Transcription kit from Promega (USA). For real-time PCR, two microliters of synthesized cDNA were utilized along with the Quantitect SYBR Green PCR Kit from Qiagen (USA). The PCR reactions were conducted using the LightCycler PCR device from Roche (Switzerland). The primers used in this study are shown in Table 1.

**Table 1:** qPCR primer sequences used in this study.

**Genes**	**Forward primer**	**Reverse primer**
TREM2	ACTACTCTGCCTGAACAC	GCTAAATATGACAGTCTTGGA
MAFB	GTGCAGGTATAAACGCGTCC	CACCTCCTGCTTAAGCTGCTC
KLF2	CCAAGAGTTCGCATCTGAAGGC	CCGTGTGCTTTCGGTAGTGGC
EPB41L3	AAAGAGGCCAAAGAGCAGCA	GCAAGCTAAGTTATTCCTCTGGTA
THEMIS2	ATGTCTTGGTTTGTCAGCGG	TCAGATCTGCCAGGCTGTAG
CD14	ACTGACTCTTGAAAACCTCG	AGCGCTAAAACTTGGAGGGT
EPB41L2	CCAGTTTGCCCCTACTCAGA	TGTCCACACCTTCTGAGTCC
GAPDH	GTCTCCTCTGACTTCAACAGCG	ACCACCCTGTTGCTGTAGCCAA

### Establishment and validation of TAMGs-related models

In this study, statistical significance for genes significantly associated with overall survival in the training set was determined using univariate Cox regression in R software, with *p*-values < 0.05 considered statistically significant. For the multivariate Cox regression analysis in the training cohort, gene expression levels (expr) and regression coefficients (coef) were utilized to construct the TAMGs risk model. The risk score (RS) for each patient was calculated using the following formula:

RS = expr_gene_1 × coef_gene_1 + expr_gene_2 × coef_gene_2 + ... + expr_gene_n × coef_gene_n.

To visualize the prognostic signatures, heatmaps were generated using the “pheatmap” package in R software. Survival curves based on TAMGs-related prognostic scores were plotted using the “survival” package. Receiver operating characteristic (ROC) curve analyses were performed using both the training and test sets, and the area under the curve (AUC) was calculated for the model using the R package “survivalROC”. The training set comprised TCGA-OV data, while the validation set consisted of GSE63885 data [22, 23].

### Western blot

For total protein extraction, RIPA lysis buffer (Beyotime, China) containing protease inhibitors was used to lyse the cells. The lysates were then denatured, and the protein samples were separated using 10% sodium dodecyl sulfate-polyacrylamide gel electrophoresis (SDS-PAGE). Subsequently, the proteins were transferred onto a polyvinylidene difluoride membrane (Millipore, USA). To block non-specific binding sites, the membrane was incubated with 5% skim milk. Primary antibodies against STK17B, E-Cadherin, Vimentin, and N-Cadherin were then added to the membrane and incubated overnight at 4°C. Afterward, the membrane was washed three times with Tris-buffered saline with Tween (TBST). Then, the membrane was incubated with horseradish peroxidase-conjugated secondary antibodies for 1 hour at room temperature. After an additional three washes with TBST, the protein bands were visualized using an electrochemiluminescence kit (Beyotime, China). Antibodies used were ab238099 (1:1000), ab98952 (1:1000), ab236975 (1:2000), and ab137321 (1:2000) (1:2000). The specific procedure can be found in the previously published publications [24–26].

### Migration assay and Transwell assay

For the wound healing assay, ovarian cancer cells were seeded in 6-well plates and allowed to reach 90% confluence before transfection with STK17B siRNA. A clean line was created in the middle of the well using a sterilized 200-μL pipette tip, and the wells were washed with PBS to remove any floating cells [27, 28]. Images of the cell migration were captured at 0 and 24 hours after creating the wound to evaluate the healing ability of the cells. In the Transwell assay, cell invasion was measured using Transwell chambers coated with Matrigel (Corning, USA). A total of 5 × 10^4^ cells suspended in 100 μL of serum-free medium were added to the upper chamber, while the bottom chamber was filled with 500 μL of Dulbecco’s modified Eagle’s medium containing 20% FBS. After 48 hours of incubation, cells that had invaded through the Matrigel and migrated to the lower surface of the membrane were fixed and stained with 0.1% crystal violet.

### Statistical analysis

All statistical analyses were performed using R software, specifically version 4.1.2. To assess differences between two independent groups, Student’s *t*-tests (unpaired, two-tailed) were utilized. For data involving more than two groups, one-way analysis of variance (ANOVA) and Kruskal-Wallis tests were applied as appropriate. Differentially expressed genes (DEGs) were identified using the “limma” R package. Mutation status in ovarian cancer patients was calculated using the “maftools” R package. The fractions of immune cell types were determined using the “CIBERSORT” R package. To explore biological differences between subtypes, GSVA (Gene Set Variation Analysis) enrichment analysis was conducted using the “GSVA” R package [29, 30]. Volcano plots and heatmaps were generated using the “ggplot2” package [31]. The significance level was set at *P* < 0.05, unless otherwise specified.

### Data availability statement

The datasets presented in this study can be found in online repositories. The names of the repository/repositories and accession number(s) can be found in the article.

## RESULTS

### Identification of TAMGs in ovarian cancer

**Figure 2:**
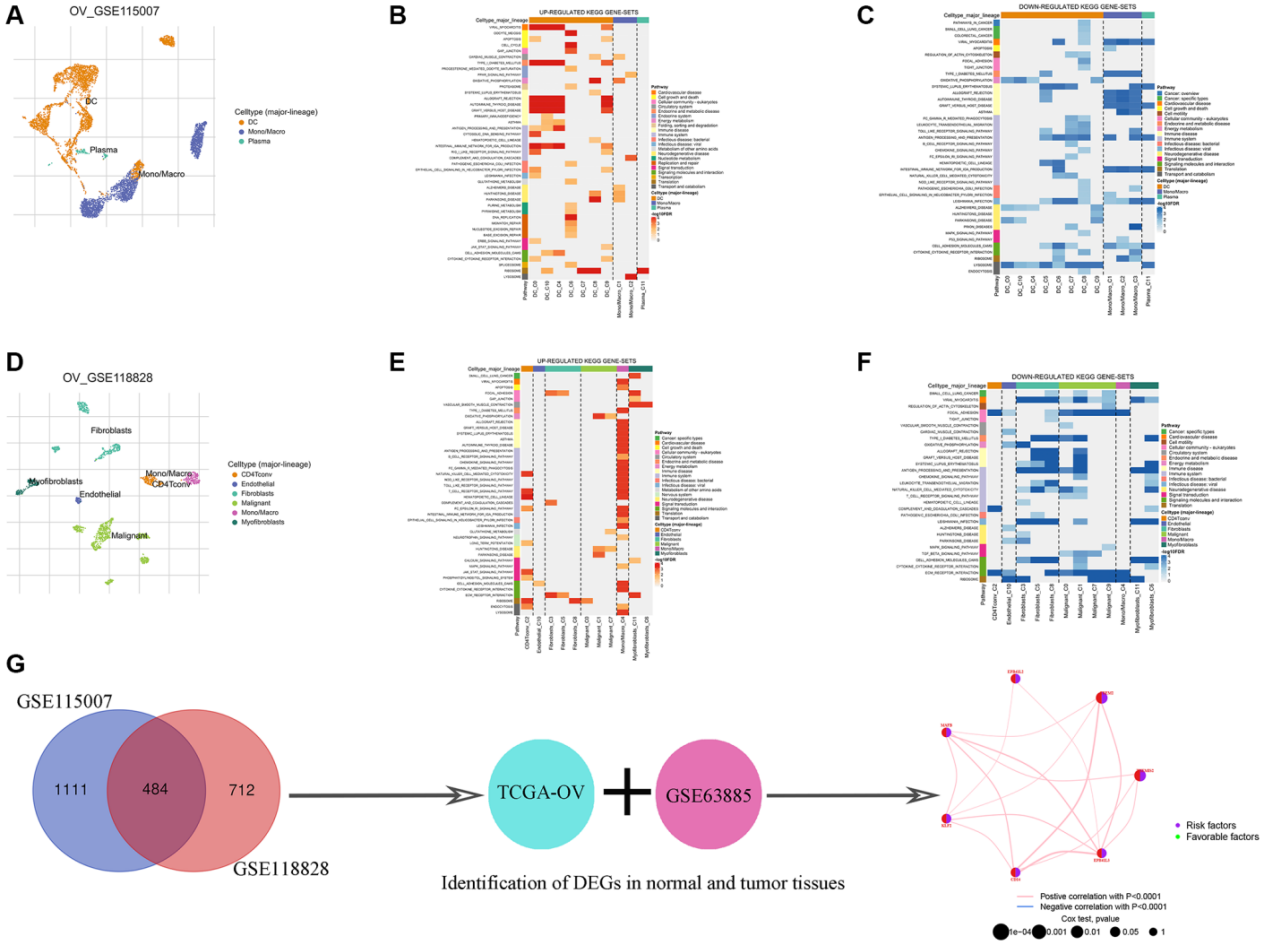
**Identification of TMAGs in ovarian cancer.** Ovarian cancer single-cell data analysis based on the GSE115007 dataset. (**A**) UMAP plots with cells colored by cell type are displayed. Heatmap showing enriched up- (**B**) or down-regulated (**C**) pathways identified based on differential genes in each cell type. Ovarian cancer single-cell data analysis based on the GSE118828 dataset. (**D**) UMAP plots with cells colored by cell type are displayed. Heatmap showing enriched up- (**E**) or down-regulated (**F**) pathways identified based on differential genes in each cell type. (**G**) Identification of key genes.

To identify potential TAMGs in ovarian cancer, we conducted an analysis of two independent single-cell sequencing datasets using the TISCH2 platform (Figure 2A, 2D). We extracted the TAMGs from these datasets and identified 484 genes that were common between the two. Functional analysis revealed that up-regulated TAMGs in ovarian cancer were predominantly associated with PPAR signaling, oxidative phosphorylation, B cell receptor signaling, chemokine signaling pathways, nod-like receptor signaling pathways, T cell receptor signaling pathways, TOLL-like receptor signaling pathways, and MAPK signaling pathways (Figure 2B, 2E). Conversely, down-regulated TAMGs were mainly related to apoptosis, allograft rejection, TOLL-like receptor signaling pathway, ECM receptor interaction, and P53 signaling pathway (Figure 2C, 2F).

To enhance the size and reliability of our study, we merged mRNA expression profiling data of ovarian cancer from the TCGA and GEO databases. Consequently, we identified 7 genes with significantly different expression levels between the normal and tumor groups, all of which were associated with ovarian cancer risk (Figure 2G; Table 2). Additionally, an important finding was the potential positive correlation between the expression levels of these genes (Figure 2G). We employed two different algorithms (EPIC and TIMER) to further validate the association of these 7 genes with TAMs, and the results demonstrated a significant correlation between them (Table 3).

**Table 2:** TMAGs with potential prognostic value.

**Id**	**HR**	**HR.95L**	**HR.95H**	***p*-value**	**km**
THEMIS2	1.165885269	1.057295	1.285629	0.002092	3.93E-06
TREM2	1.16430442	1.044744	1.297547	0.005928	3.03E-06
EPB41L2	1.130252781	1.0205	1.25181	0.018808	9.72E-05
MAFB	1.159319928	1.022011	1.315076	0.021534	8.00E-05
KLF2	1.124067024	1.011062	1.249702	0.030504	0.012992
CD14	1.131449513	1.010191	1.267263	0.032739	5.48E-05
EPB41L3	1.113665904	1.006001	1.232853	0.037958	8.83E-06

**Table 3:** Correlation Analysis of Genes and TAMs.

**Genes**	**Cancer**	**Method**	**Correlation**	***P***
THEMIS2	OC	EPIC	0.731	<0.05
		TIMER	0.151	<0.05
TREM2	OC	EPIC	0.34	<0.05
		TIMER	0.334	<0.05
EPB41L2	OC	EPIC	0.171	<0.05
		TIMER	0.246	<0.05
MAFB	OC	EPIC	0.401	<0.05
		TIMER	0.534	<0.05
KLF2	OC	EPIC	0.158	<0.05
		TIMER	0.209	<0.05
CD14	OC	EPIC	0.898	<0.05
		TIMER	0.321	<0.05
EPB41L3	OC	EPIC	0.625	<0.05
		TIMER	0.408	<0.05

### Validation of expression levels of key TAMGs

**Figure 3:**
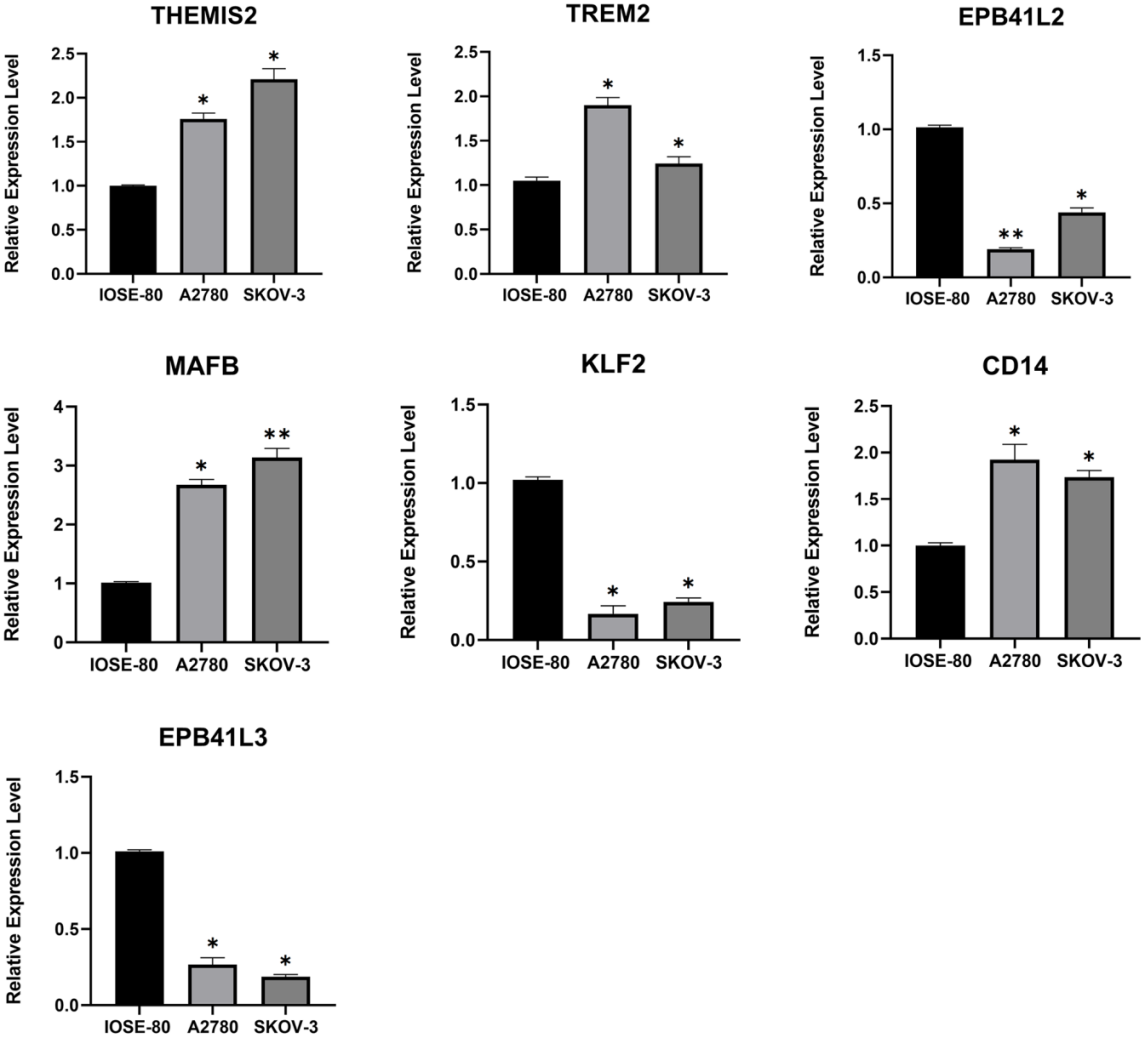
**The mRNA expression levels of 7 TMAGs.** Expression data were normalised to the reference genes (GAPDH), and are presented relative to the “calibrator” (IOSE-80). ^*^P < 0.05, ^**^P < 0.01, and ^***^P < 0.001.

We identified 7 key TAMGs in OC based on single-gene sequencing data. To validate their expression levels, we utilized the GEPIA2 database, which confirmed that their expression levels were significantly dysregulated in tumor patients (Supplementary Figure 1). Furthermore, immunohistochemistry results provided additional confirmation, demonstrating significant changes in protein levels of these TAMGs in tumor tissues (Supplementary Figure 2). To further investigate their mRNA expression levels, we performed PCR experiments on common OC cell lines. The results were consistent with the findings from the public data analysis. Specifically, genes THEMIS2, TREM2, MAFB and CD14 exhibited significantly higher expression levels in OC, while genes EPB41L2, KLF2, and EPB41L3 showed significantly lower expression levels in OC (Figure 3).

### TAMGs-based identification of ovarian cancer subtypes

**Figure 4:**
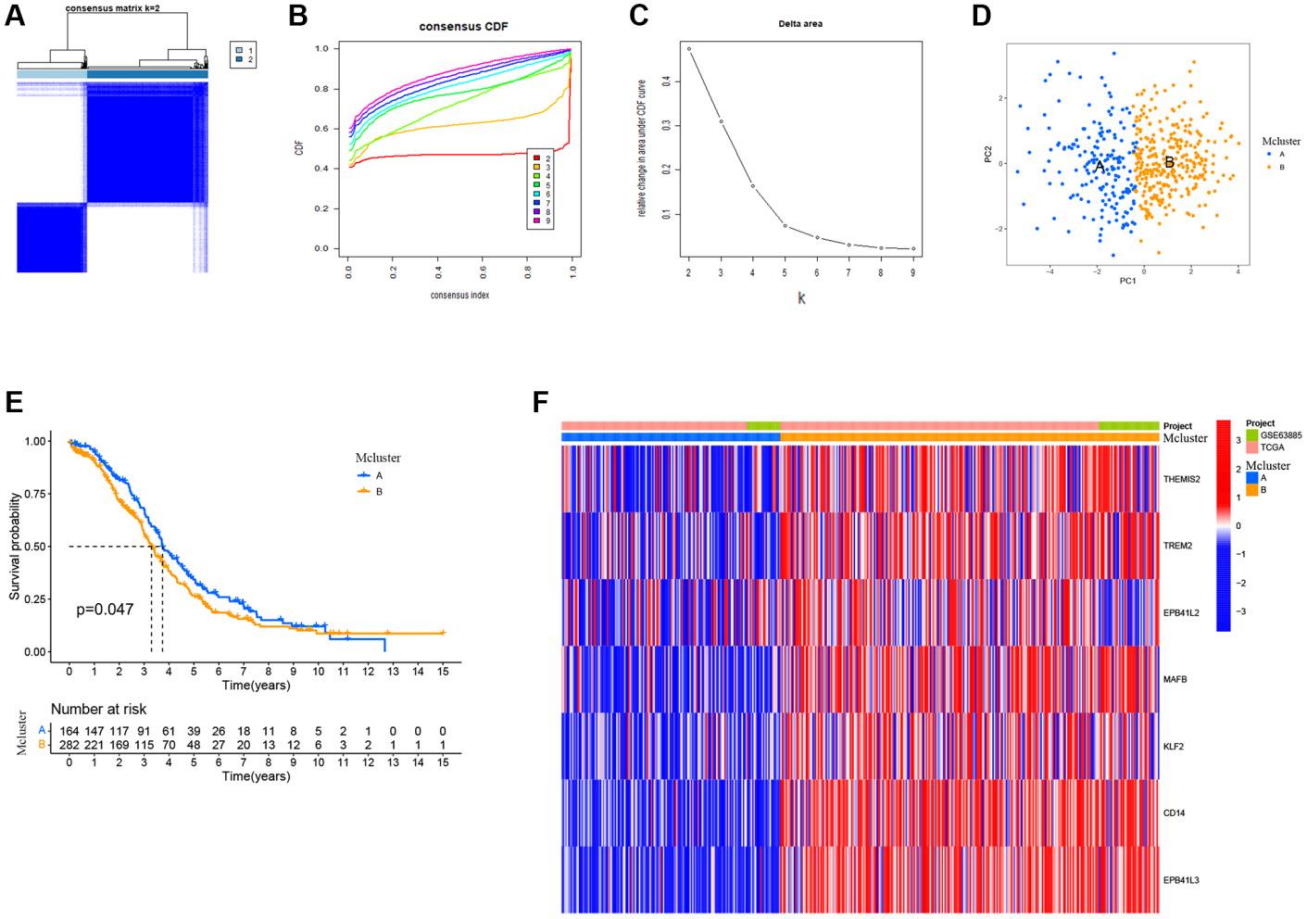
**TMAGs-based identification of ovarian cancer subtypes.** (**A**) Patients in two cohorts were grouped into two clusters according to the consensus clustering matrix (k = 2). (**B**) CDF curves of the consensus score (k = 2–9) in the two cohorts. (**C**) Relative change in the area under the CDF curve (k = 2–9) in the two cohorts. (**D**) PCA plot for OSCC patients based on the Mcluster. (**E**) Kaplan–Meier survival analyses of the patients with Mcluster A and Mcluster B. (**F**) Heatmap showing the distribution of expression of genes in the model.

In order to gain a more comprehensive insight into the prospective applicability of TAMGs in the context of OC, we conducted a comprehensive analysis of pivotal TAMGs, contingent upon their expression profiles with the patient. This analysis harnessed data sourced from TCGA-OC and GSE63885. By increasing the clustering variable (k) from 2 to 9, we observed that when k = 2, the intra-group correlation was the highest and the inter-group correlation was the lowest. This indicates that OC patients can be effectively divided into two distinct subtypes (Figure 4A–4C). Furthermore, the expression levels of TAMGs exhibited significant differentiation between these two subtypes, as demonstrated by the PCA plot (Figure 4D). Kaplan-Meier analysis revealed that patients in the MCluster A group had a better prognosis compared to those in the MCluster B group (*P* = 0.047; Figure 4E). Consistently, the heat map analysis showed significantly lower levels of TAMG expression in MCluster A compared to MCluster B (Figure 4F).

Further comparison between the two subtypes revealed that the MCluster B group was enriched with various immune cells, including B cells, CD4 T cells, CD8 T cells, dendritic cells, immature B cells, macrophages, mast cells, monocytes, natural killer cells, and others (Supplementary Figure 3A). Additionally, functional enrichment analysis identified significant functional differences between the subtypes. Signaling pathways such as B cell receptor signaling, T cell receptor signaling, neurotrophin signaling, TOLL-like receptor signaling, ERBB signaling, chemokine signaling, and FC epsilon RI signaling were found to be significantly enriched in the MCluster B group (Supplementary Figure 3B).

### Construction and validation of a prognostic signatures model of TAMGs

**Figure 5:**
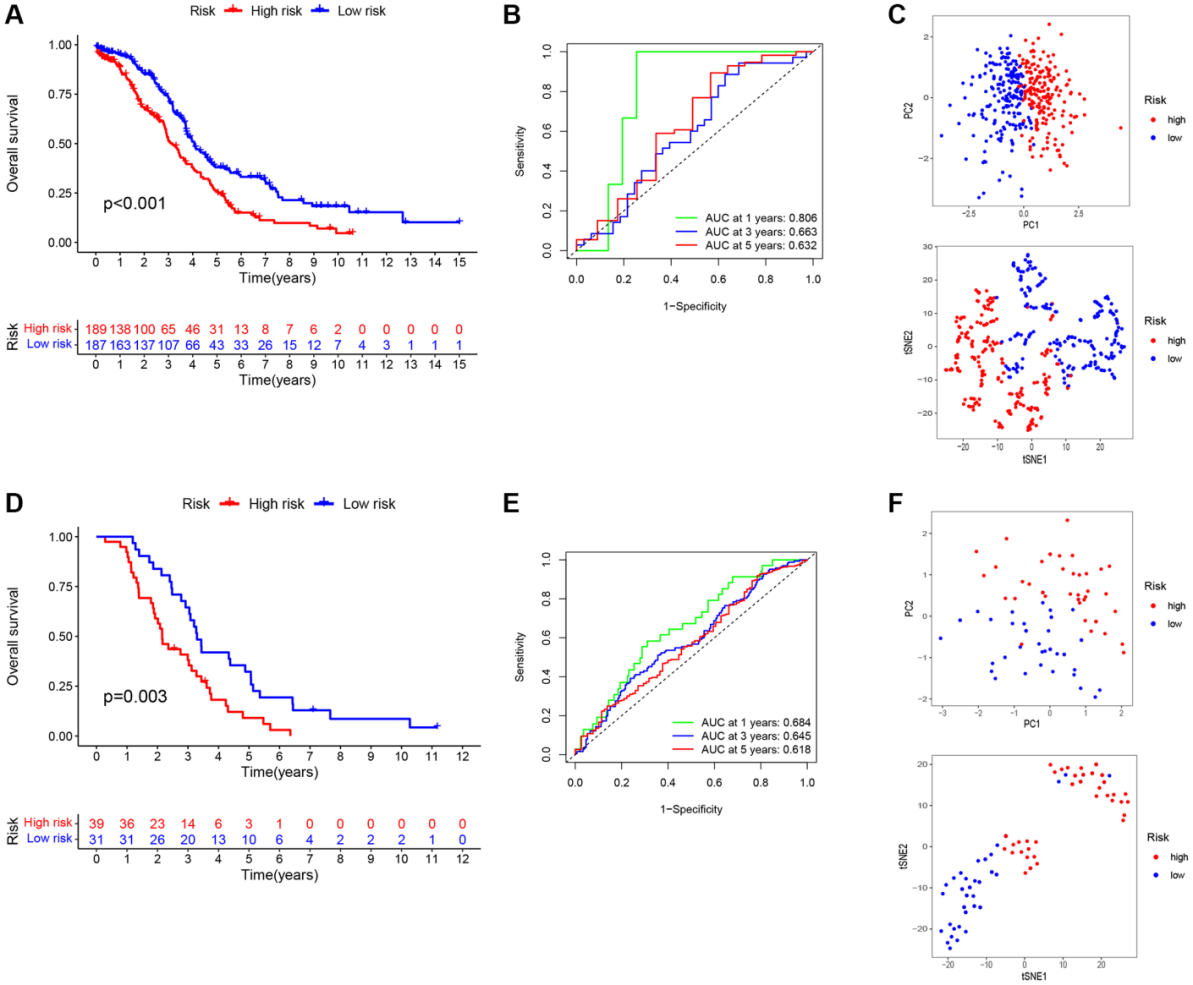
**Construction and validation of a prognostic signatures model of TMAGs.** (**A**) Kaplan-Meier survival curves for the training set. (**B**) ROC analysis of the training set. (**C**) PCA (above) and t-SNE (below) analysis of the training set. (**D**) Kaplan-Meier survival curves for the validation set. (**E**) ROC analysis of the validation set. (**F**) PCA (above) and t-SNE (below) analysis of the validation set.

We formulated a prognostic model that incorporates signatures based on TAMGs to assess their prognostic relevance in OC. In both the training cohort (*P* < 0.001; Figure 5A) and the validation cohort (*P* = 0.003; Figure 5D), a high RS exhibited a statistically significant association with an unfavorable prognosis. The patient data for the training group were sourced from TCGA-OV, while data for the validation group were derived from the GSE63885 dataset.

To evaluate the specificity and accuracy of the model, time-dependent ROC curves were generated, showing AUC values of 0.806, 0.663, and 0.632 for the 1-, 3-, and 5-year predictions, respectively, for the three risk signatures (Figure 5B). The validation model exhibited good predictive performance for this risk signatures at 1-, 3-, and 5-years, as depicted in Figure 5E. Furthermore, principal component analysis (PCA) and t-distributed stochastic neighbor embedding (t-SNE) analyses demonstrated that our constructed feature model could effectively differentiate ovarian cancer patients based on their RS (Figure 5C, 5F).

### TAMGs-based RS to predict the immune landscape of ovarian cancer patients

**Figure 6:**
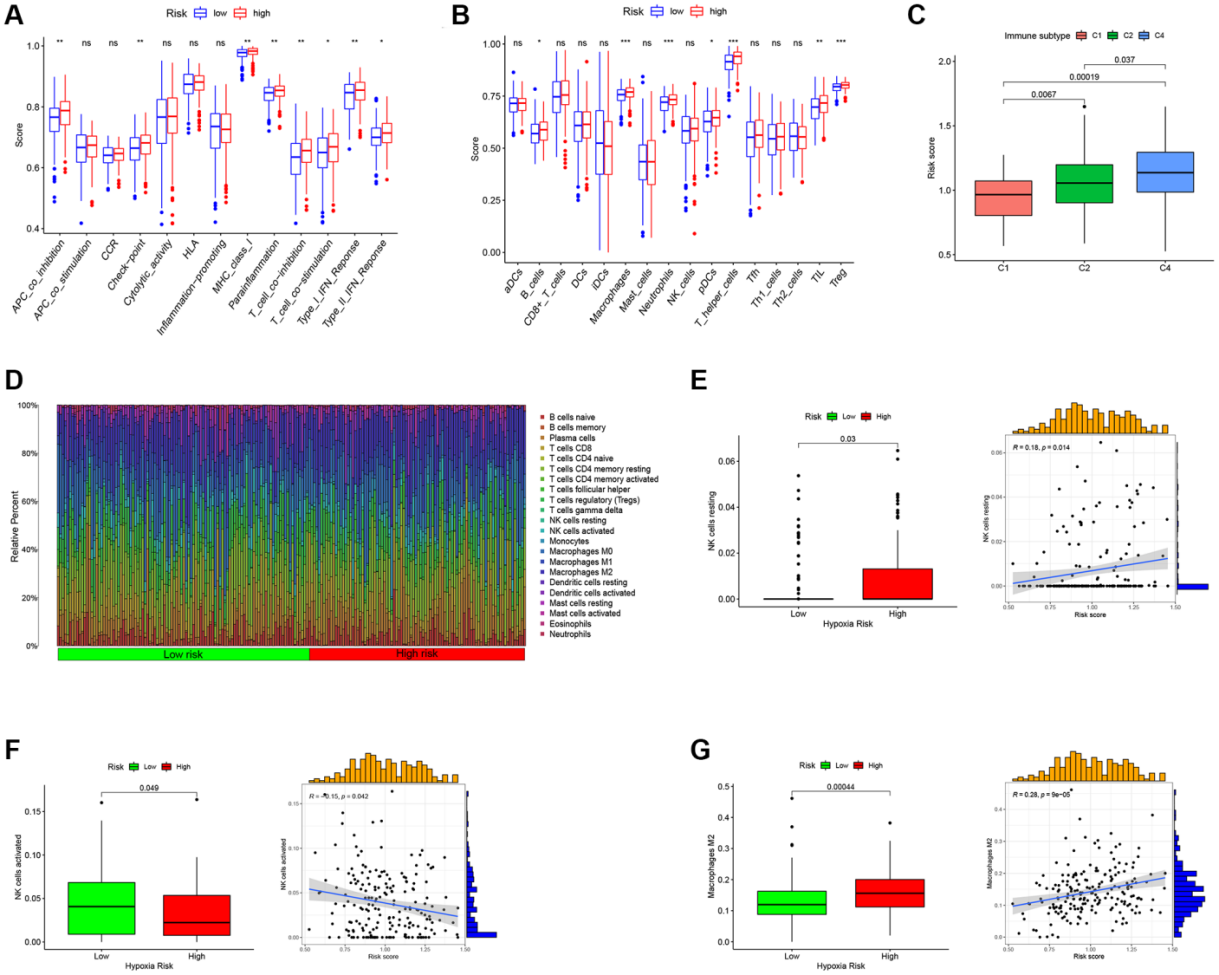
**Predict the immune landscape of ovarian cancer patients.** (**A**) Analysis of immune function between high- and low-RS. (**B**) Immune cell enrichment analysis between high- and low-RS. (**C**) The relationship between RS and immune subtypes. (**D**) The bar plot showing the proportion of infiltrated immune cells calculated by the CIBERSORT algorithm. (**E**) RS has a positive correlation with NK cells resting. (**F**) RS has a negative correlation with NK cells activated. (**G**) RS has a positive correlation with Macrophages M2.

In our subsequent investigation, we focused on exploring the immune function (Figure 6A) and immune cell enrichment (Figure 6B) differences between the high- and low-RS groups using the ssGSEA algorithm. Our findings revealed that the high RS group exhibited higher immune cell enrichment and displayed increased activity in immune-related functional pathways. Notably, functional pathways such as APC co-inhibition, MHC class I, T cell co-inhibition, Type I IFN Response, Type II IFN Response, B cell, Neutrophils, pDCs, TIL, and Treg showed significant enrichment in the high RS group. We also observed a correlation between RS and the immune classification of ovarian cancer patients, with the high RS group being categorized as C4 (lymphocyte depletion) type, while the low RS group belonged to the C1 (wound healing) type (Figure 6C). Moreover, an analysis of immune cell enrichment in the high and low RS groups (Figure 6D) further supported the differences in immune cell composition between the two groups. Furthermore, we found positive correlations between RS and resting NK cells (Figure 6E) as well as macrophage M2 (Figure 6G), indicating a potential association between RS and these immune cell subsets. Conversely, there was a negative correlation between RS and activated NK cells (Figure 6F).

### RS-Based prediction of immunotherapy response

**Figure 7:**
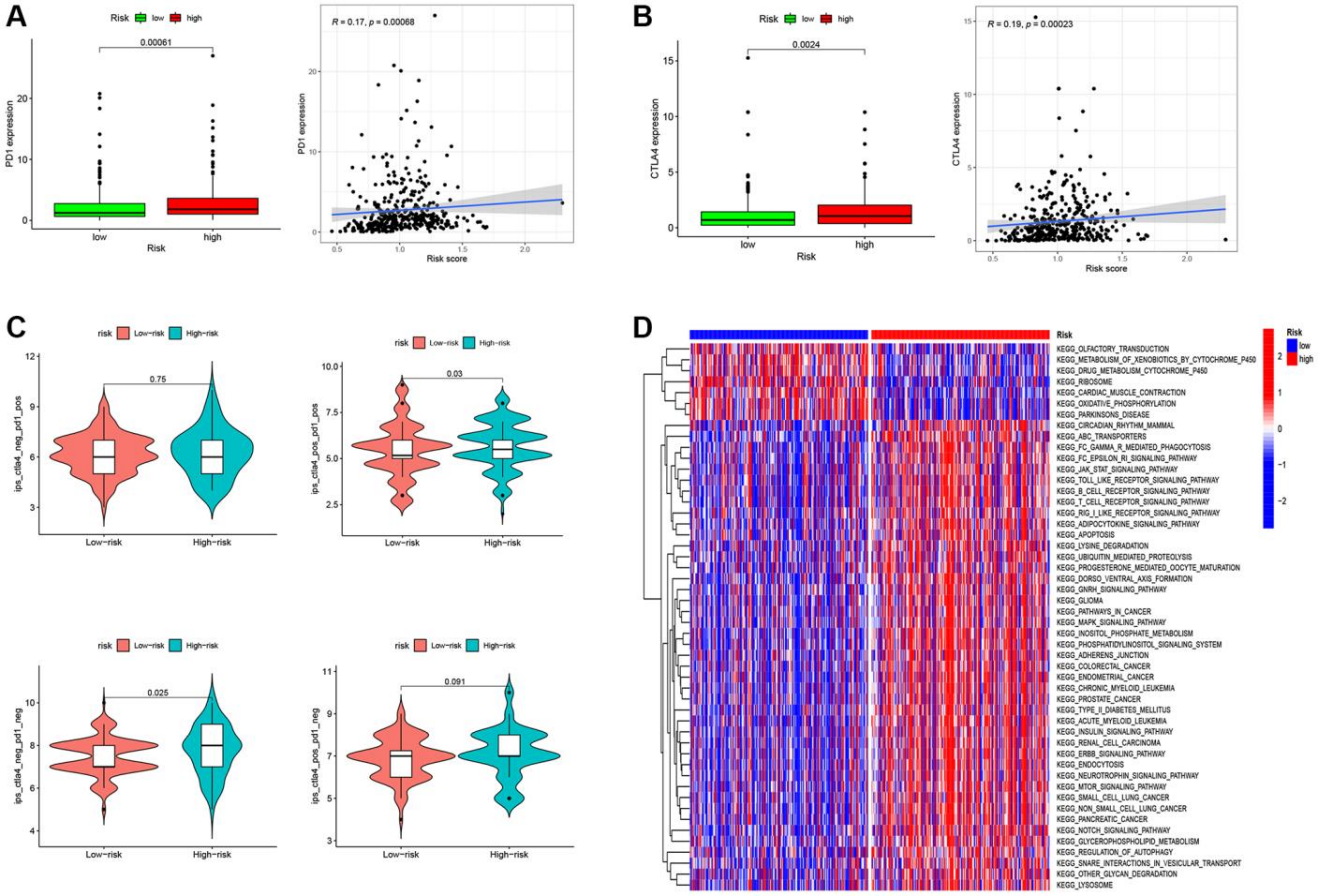
**RS-Based prediction of immunotherapy response.** (**A**) Correlation analysis between RS and PD1. (**B**) Correlation analysis between RS and CTLA4. (**C**) RS-Based Prediction of Immunotherapy Response. (**D**) Functional enrichment analysis between high- and low-RS groups. ^*^P < 0.05, ^**^P < 0.01, and ^***^P < 0.001.

In the context of OC patients, the prognostic implications of TAMGs-related RS exhibit marked significance and exert discernible effects on the TME. Our investigation delves deeper into the potential of RS as a predictive marker for the efficacy of immunotherapy. To this end, we conducted a comprehensive analysis of the expression profiles of PD1 and CTLA4, which represent pivotal immune checkpoint genes. Notably, we observed that the high RS group demonstrated elevated expression levels of these genes, and a consistent, positive correlation between them was established, as visually represented in Figure 7A, 7B.

In our endeavor to assess the role of RS in prognosticating immunotherapeutic response, we undertook an examination of Immune Prognostic Score (IPS) data obtained from ovarian cancer patients, employing the TCIA database (Figure 7C). Our findings unveil a statistically significant elevation in the median IPS value for patients within the high RS group as compared to their low RS counterparts. This outcome signifies a heightened probability of favorable responsiveness to immunotherapy in the cohort with an elevated RS.

Functional enrichment analysis provided further insights into the biological pathways associated with high and low RS groups. The low RS group showed enrichment in functional pathways such as Olfactory transduction, Metabolism of xenobiotics by cytochrome p450, Drug metabolism cytochrome p450, Ribosome, cardiac muscle contraction, and Oxidative phosphorylation. In contrast, the high RS group exhibited substantial enrichment of functional pathways related to cancer and immune cells. These pathways included circadian rhythm mammals, FC gamma r mediated phagocytosis, FC epsilon ri signaling pathways, JAK-STAT signaling pathways, Toll-like receptor signaling pathways, B cell receptor signaling pathways, T cell receptor signaling pathways, Apoptosis, Gnrh signaling pathways, Cancer pathways, MAPK signaling pathways, Neurotrophin signaling pathways, MTOR signaling pathways, Notch signaling pathways, and more (Figure 7D). These findings suggest that the high RS group may possess a more active and immunogenic TME, potentially contributing to increased responsiveness to immunotherapy.

### THEMIS2 is a potential target in ovarian cancer patients and is associated with EMT

**Figure 8:**
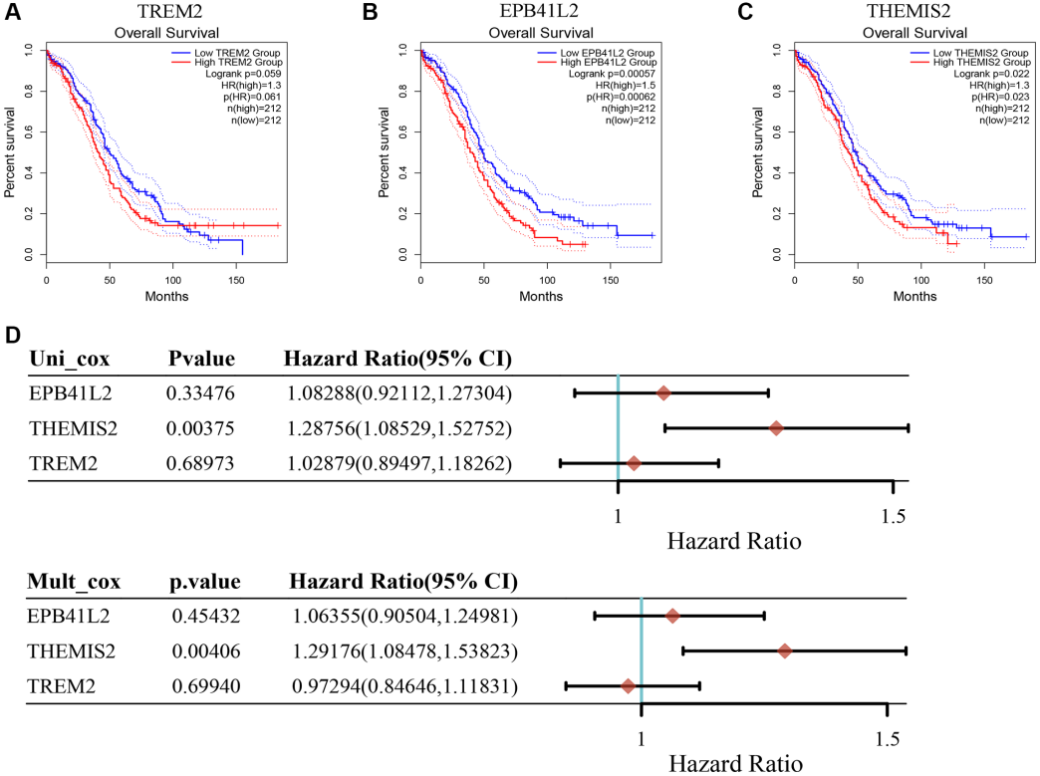
**The relationship between key genes in TMAGs model and prognosis of OC.** (**A**) TREM was not significantly associated with the prognosis of OC patients. (**B**) High expression of EPB41L2 was associated with poor prognosis in OC patients. (**C**) High expression of THEMIS2 was associated with poor prognosis in OC patients. (**D**) Univariate and multivariate COX analysis results showed that A was an independent prognostic factor for OC patients.

To further illuminate the potential implications of the three genes incorporated into our model in the context of OC, we conducted supplementary investigations that encompassed both bioinformatics analyses and *in vitro* experiments. Our findings underscore that heightened expression levels of EPB41L2 (*P* = 0.00062; Figure 8B) and THEMIS2 (*P* = 0.023; Figure 8C) were significantly associated with an adverse prognosis among OC patients. In contrast, TREM2 (*P* = 0.061; Figure 8A) did not exhibit a noteworthy correlation with patient prognosis. Moreover, the results from univariate and multivariate Cox analyses lent support to the notion that THEMIS2 might function as an independent prognostic factor in the realm of OC, as visually represented in Figure 8D.

**Figure 9:**
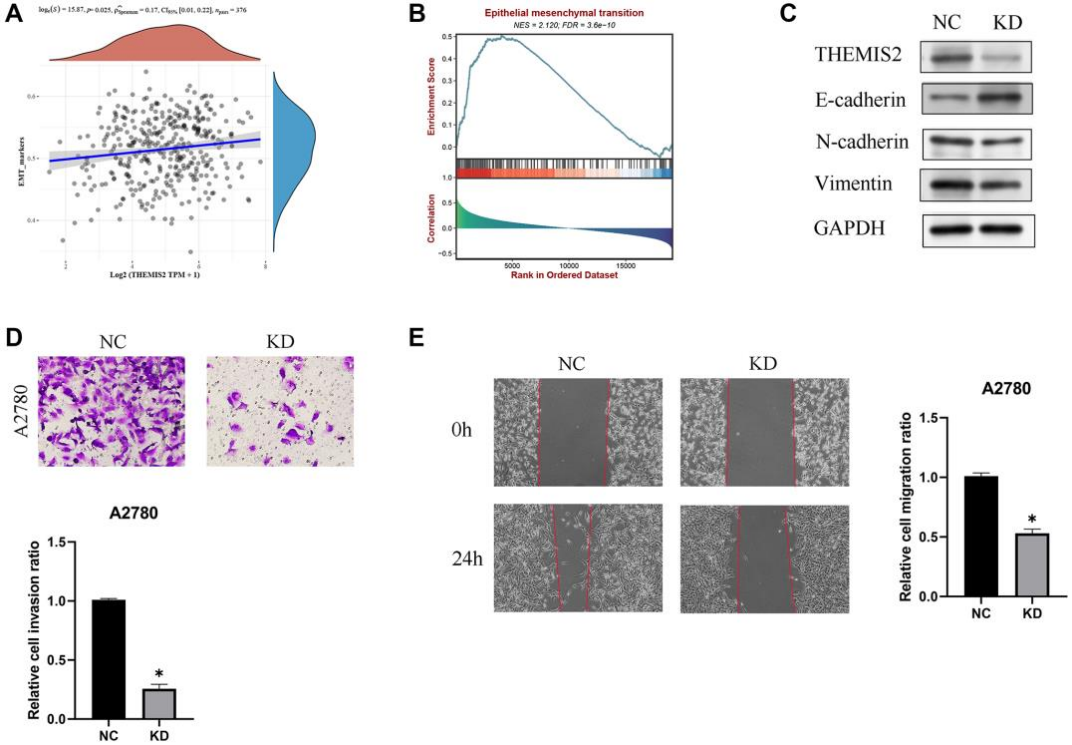
**Knockdown of THEMIS2 in OC cells inhibits cell migration and invasion.** (**A**) THEMIS2 was positively correlated with EMT-related markers. (**B**) EMT-related pathways were significantly enriched in highly expressed THEMIS2. (**C**) The expression of EMT-related proteins was significantly affected after THEMIS2 knockout. (**D**) The invasion ability of OC cells was significantly reduced after THEMIS2 knockdown. (**E**) The migratory ability of OC cells was significantly reduced after THEMIS2 knockdown.

Subsequent research unveiled a significant positive correlation between THEMIS2 expression and markers associated with epithelial-mesenchymal transition (EMT), suggesting its involvement in the EMT pathway (Figure 9A, 9B). To investigate this further, we performed *in vitro* experiments wherein THEMIS2 was silenced in ovarian cancer cells. The knockdown of THEMIS2 resulted in the suppression of N-cadherin and vimentin expression, while promoting E-cadherin expression, indicating a reversal of the EMT process in these cells (Figure 9C). Moreover, the inhibition of THEMIS2 significantly impeded the migration and invasion of ovarian cancer cells (Figure 9D, 9E). These findings provide compelling evidence for the involvement of THEMIS2 in EMT within the context of ovarian cancer. The suppression of THEMIS2 expression not only disrupts EMT-associated molecular markers but also hampers the migratory and invasive capabilities of ovarian cancer cells. These results shed light on the functional role of THEMIS2 in EMT and suggest its potential as a therapeutic target for inhibiting EMT-driven processes in ovarian cancer.

## DISCUSSION

OC is a highly lethal gynecological tumor with a low 5-year survival rate, highlighting the urgent need for new biomarkers and therapeutic strategies [31–33]. In the tumor microenvironment of OV, TAMs are among the most abundant immune cells. TAMs can originate from circulating monocytes that are recruited to the tumor site by chemotactic signals released by cancer cells and the surrounding stroma. Once at the tumor site, TAMs undergo functional polarization and can exhibit both pro-tumor and anti-tumor properties, depending on the specific microenvironmental cues. TAMs, as important components of the OC microenvironment, play a significant role in the regulation of OC progression, invasion, metastasis, and drug resistance. Studies have shown that TAMs contribute to the adaptive immune response and antitumor development during chemotherapy in OC [34]. Wang et al. showed that targeting TAM is a promising strategy for altering the immunosuppressive tumor microenvironment and improving cancer immunotherapy [35]. In addition, TAM recruitment and activation have been reported to be closely associated with OV development and metastasis [36].

Within the context of OC, TAMs frequently adopt an M2-like phenotype, which is notably associated with immunosuppressive and tumor-promoting effects. These M2-like TAMs release anti-inflammatory cytokines, such as interleukin-10 (IL-10) and transforming growth factor-beta (TGF-β), which collectively contribute to the dampening of the immune response against cancer cells [37–40]. Additionally, they play a pivotal role in fostering angiogenesis, tissue remodeling, and the creation of an immunosuppressive milieu within the tumor. Consequently, the targeting of TAMs in the context of OC has emerged as an active and promising realm of investigation, aimed at the formulation of novel therapeutic strategies.

In this article, we performed a comprehensive analysis of TAMGs in OC patients. Initially, we screened TAMGs using two independent single-cell datasets from OC patients. We then validated these TAMGs by selecting those associated with OC prognosis and exhibiting dysregulated expression in OC-related genes. Ultimately, we identified seven key TAMGs: THEMIS2, TREM2, EPB41L2, MAFB, KLF2, CD14, and EPB41L3. To further confirm the expression of these TAMGs, we verified their protein expression levels through WB and mRNA expression levels through PCR experiments. Our results demonstrated consistent dysregulation of these TAMGs in OC patients, supporting their potential significance in the disease.

Based on the expression patterns of these seven TAMGs, we were able to effectively categorize OC patients into two subtypes: MCluster A and MCluster B. Patients belonging to MCluster A exhibited a better prognosis compared to MCluster B. However, we observed that MCluster A patients had lower immune cell infiltration enrichment and were less sensitive to immunotherapy compared to MCluster B patients. Moreover, we developed a prognostic feature model using a scoring system consisting of three genes (EPB41L2, THEMIS2, and TREM2). The accuracy of this model was validated using independent external data, further supporting its potential clinical utility. Notably, patients with higher risk scores in the model had a poorer prognosis. Another significant finding of our study was the predictive value of TAMGs-based risk scores for immunotherapy response in OC patients. Patients characterized by higher RS demonstrated heightened sensitivity to immunotherapeutic interventions, suggesting that TAMGs may serve as prospective predictive biomarkers for the assessment of immunotherapeutic outcomes. While prognostic models in the context of OC have received considerable research attention, the development of predictive models for characterizing TAMs in OC remains a notably unresolved aspect [41–44]. This article offers a valuable source of information that can potentially enhance the understanding of TAMs in OC and provide a foundation for further study in this area.

Moreover, our *in vitro* experiments revealed potential key genes in the model. We observed significant overexpression of THEMIS2 in OC, which was correlated with poorer prognosis. Further investigations demonstrated that THEMIS2 was associated with EMT, and silencing THEMIS2 in OC cells resulted in a significant inhibition of cell migration and proliferation. In a recent study conducted by Huang et al., it was discovered that the expression of THEMIS2 exhibited a noteworthy increase in breast and ovarian cancer stem cell lines [45]. The researchers identified THEMIS2 as a novel regulator of cancer stemness and chemoresistance by disrupting the interaction between PTP1B and p-MET, thus promoting MET signaling in cancer cells. These findings suggest that THEMIS2 could serve as a promising target in OC patients, offering potential therapeutic value.

These findings collectively highlight the importance of TAMGs in OC prognosis, immunotherapy response, and the underlying tumor microenvironment. Further studies are needed to validate these findings in clinical settings and explore the molecular mechanisms underlying the observed associations.

## Supplementary Materials

Supplementary Figures
