# Supplementary material for: Signatures of tumor-associated macrophages correlate with treatment response in ovarian cancer patients
Source: Aging (Albany NY). 2024 Jan 3;16(1):207–25. doi: 10.18632/aging.205362 (PMC10817412; doi:10.18632/aging.205362)
Supplement: Supplementary Figures [file aging-16-205362-s001.pdf]

SUPPLEMENTARY FIGURES

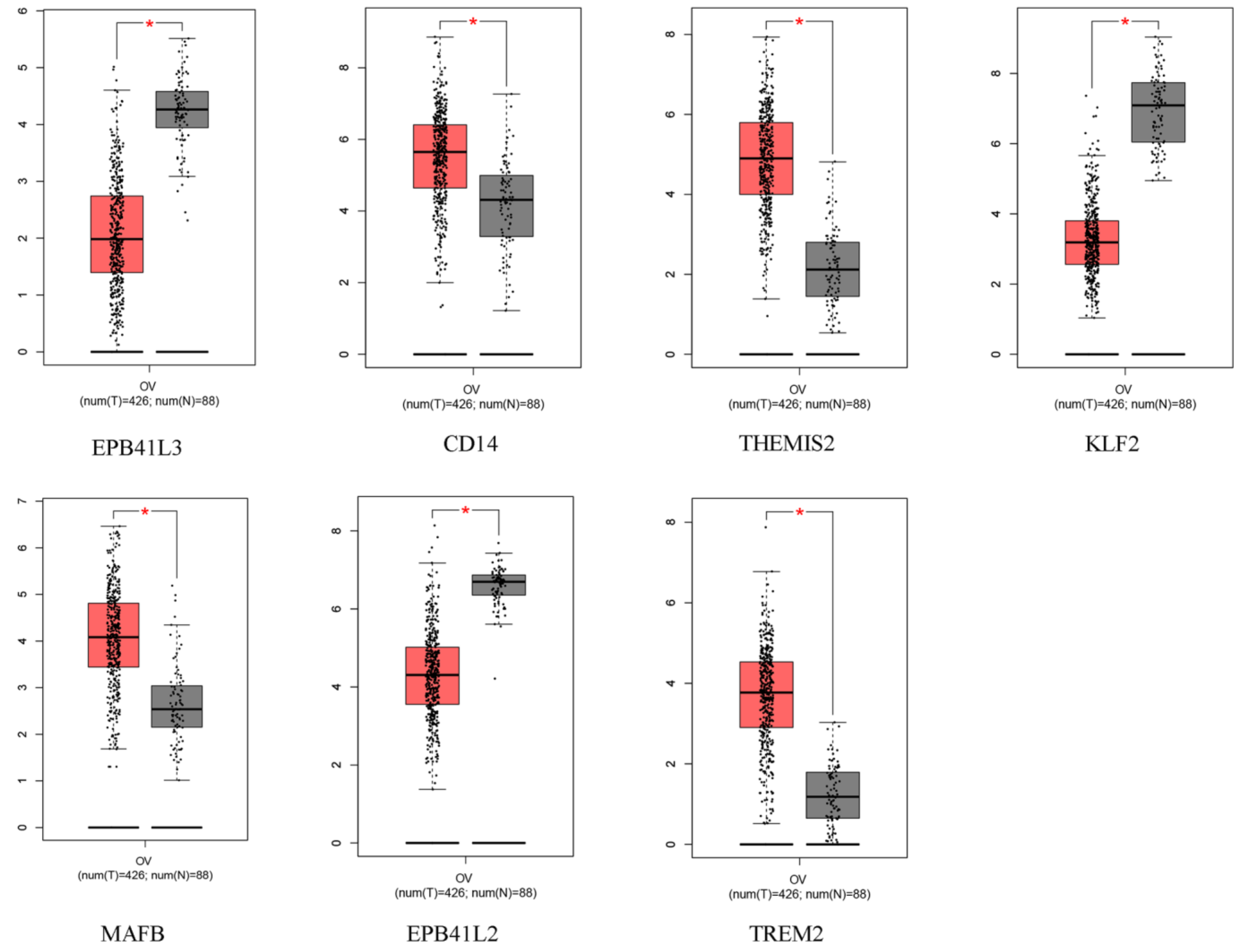

Supplementary Figure 1. Gene expression levels of GEPIA2-based key TMAGs in ovarian cancer and normal.

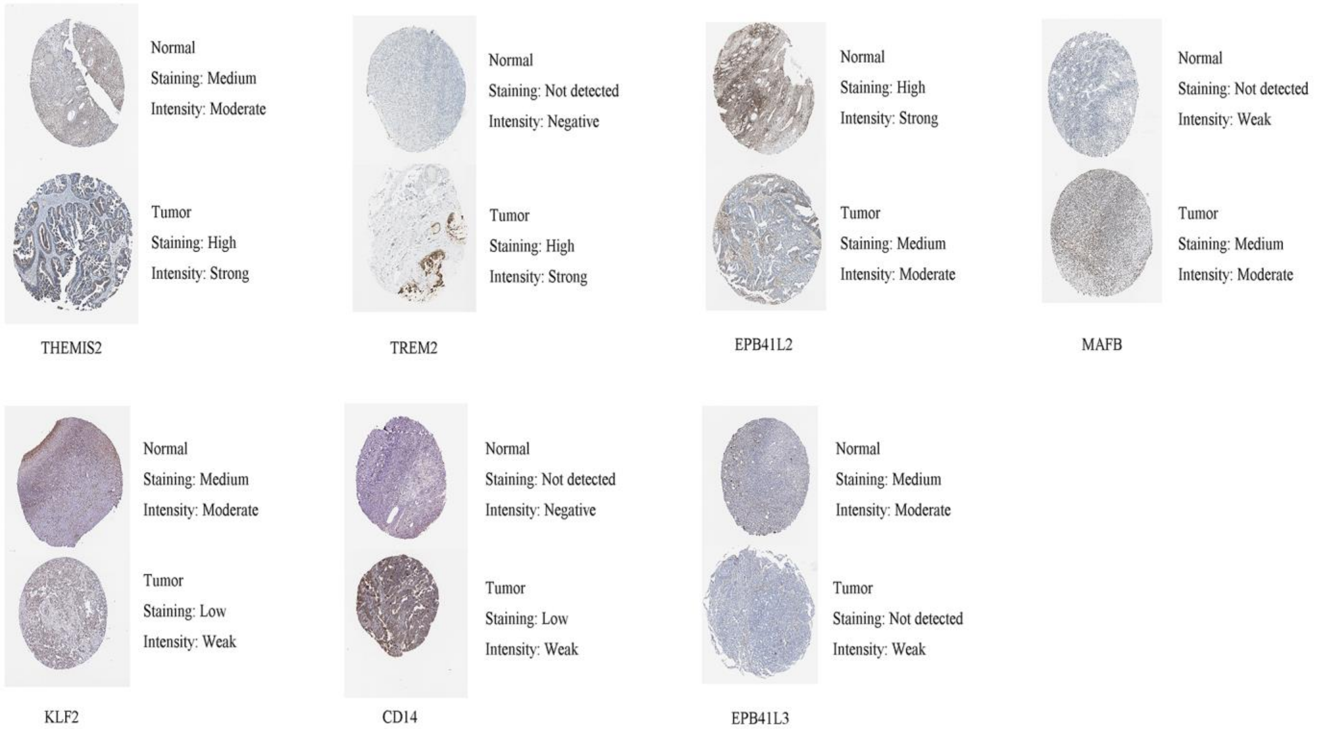

Supplementary Figure 2. Key TMAGs protein expression levels in ovarian cancer and normal. Figure modified from HPA.

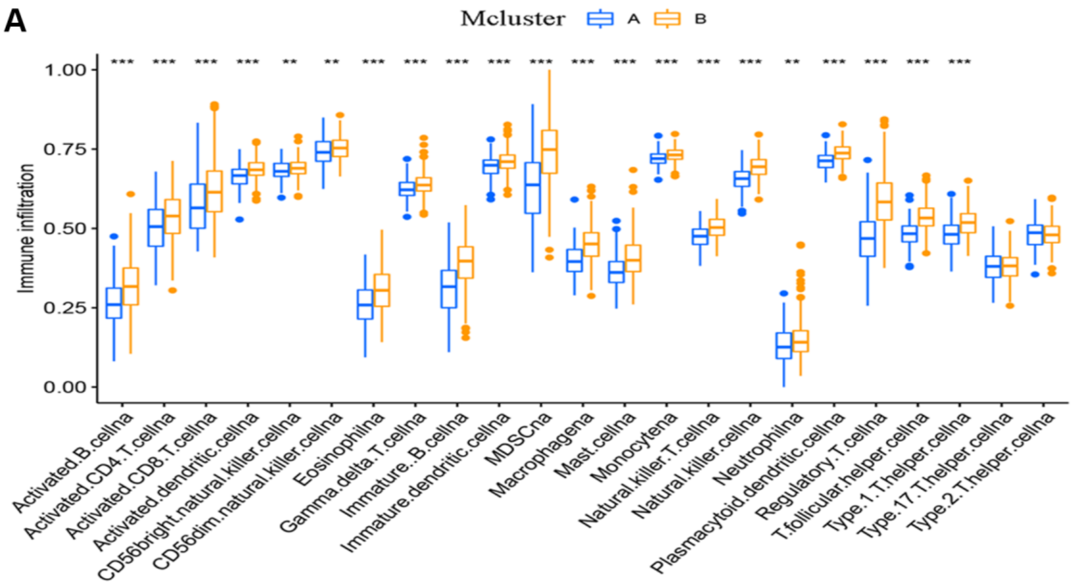

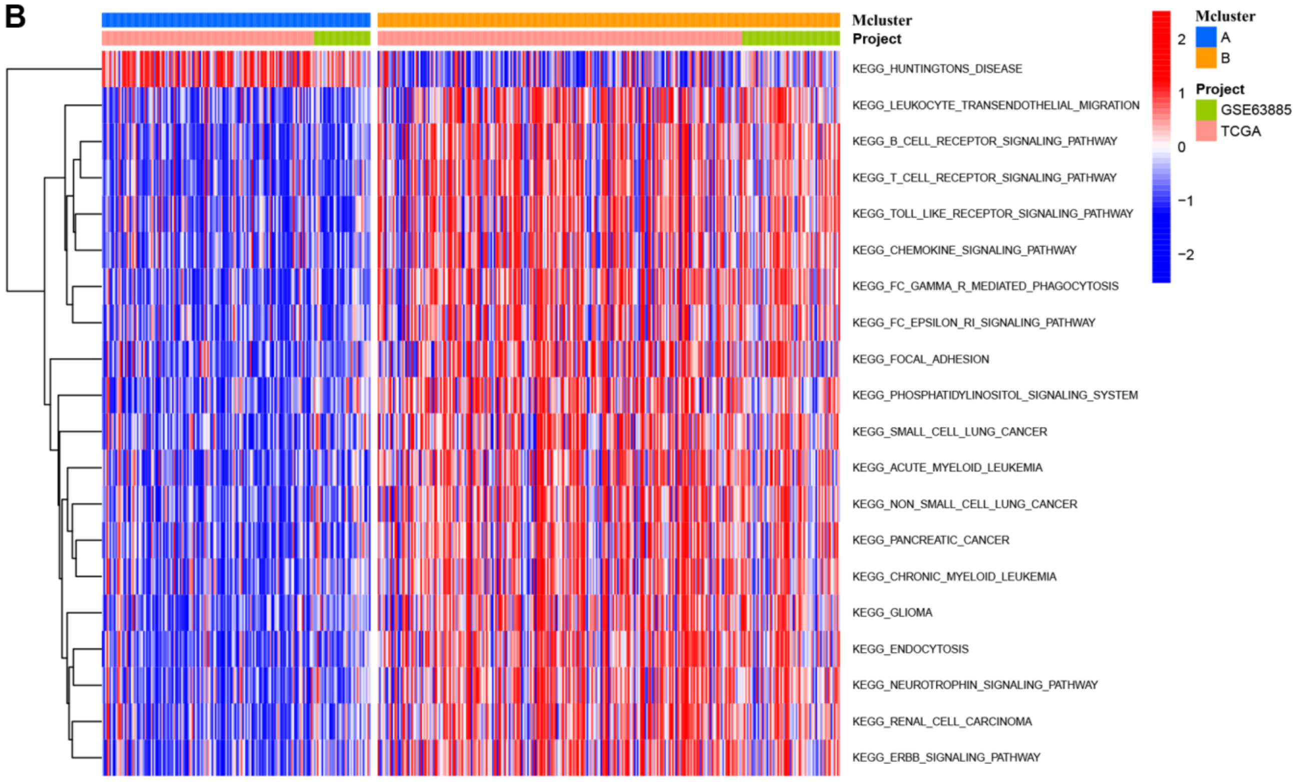

Supplementary Figure 3. Differential identification between subtypes. (**A**) Analysis of immune infiltrating cells between MCluster A and MCluster B. (**B**) Heat mapping was used to visualize the biological process by GSVA analysis in the 2 clusters. $^{*}P < 0.05$, $^{**}P < 0.01$, and $^{***}P < 0.001$.
